# The effect of fascia iliaca block on postoperative pain and analgesic consumption for patients undergoing primary total hip arthroplasty: a meta-analysis of randomized controlled trials

**DOI:** 10.1186/s13018-021-02585-1

**Published:** 2021-07-09

**Authors:** Wenli Dai, Xi Leng, Xiaoqing Hu, Jin Cheng, Yingfang Ao

**Affiliations:** 1 Institute of Sports Medicine, Beijing Key Laboratory of Sports Injuries, Peking University Third Hospital, 49 North Garden Road, Haidian District, Beijing, 100191 People’s Republic of China; 2 Medical Imaging Center, The First Affiliated Hospital of Guangzhou University of Chinese Medicine, 16 Jichang Road, Baiyun District, Guangzhou, Guangdong People’s Republic of China

**Keywords:** Fascia iliaca block, Postoperative pain, Analgesic consumption, Total hip arthroplasty, Meta-analysis

## Abstract

**Background:**

The primary aim of this systematic review and meta-analysis was to compare postoperative pain, analgesic consumption, and complications after fascia iliaca block (FIB) versus control for patients undergoing primary total hip arthroplasty (THA). Second, we compared the outcomes of FIB versus placebo. Finally, we sought to evaluate pain and analgesic consumption after preoperative and postoperative FIB.

**Methods:**

We performed a systematic literature search in MEDLINE, Embase, Scopus, Web of Science, Google Scholar, ClinicalTrials.gov, and CENTRAL through February 2021 to identify randomized controlled trials (RCTs) that evaluated the efficacy of FIB versus control for patients undergoing primary THA. All analyses were conducted on intent-to-treat data with a random-effects model.

**Results:**

Twelve RCTs with a total of 815 patients were included. There was no difference in postoperative pain (P = 0.64), analgesic consumption (P = 0.14), or complication rate (P = 0.99) between FIB and control groups. Moreover, no difference in postoperative pain (P = 0.26), analgesic consumption (P = 0.06), or complication rate (P = 0.71) was found between FIB and placebo. Moreover, sensitivity analysis suggested that no significant difference in postoperative pain, analgesic consumption, or complication rate was present between FIB and control in studies that used preoperative and postoperative FIB.

**Conclusion:**

FIB was not found to be superior to placebo or various anesthetic techniques for patients undergoing primary THA, as measured by postoperative pain, analgesic consumption, and complications.

**Supplementary Information:**

The online version contains supplementary material available at 10.1186/s13018-021-02585-1.

## Introduction

Postoperative pain is a significant concern for patients undergoing primary total hip arthroplasty (THA) [1]. Patient outcomes, including increased patient satisfaction, early participation in physical therapy, and a faster return to self-care, are influenced by postoperative pain control [2]. Furthermore, untreated acute pain is a predictor of chronic pain and disability, which negatively impacts quality of life [3]. Numerous economic benefits have also been found with improved postoperative pain control, including earlier discharge, less assistance to ambulate, decreased opioid use and opioid-related complications, and decreased thirty-day readmission rates, which are essential for avoiding financial penalties for readmissions [4, 5]. To help control pain, patients can receive a multimodal drug regimen, ice, and physical therapy. Peripheral nerve blocks are another option to help control pain postoperatively. Although there is currently little evidence for routine use of nerve blocks after THA, there have been several reports of the use of nerve blocks to decrease pain after total knee and shoulder arthroplasty [6, 7].

The hip joint is innervated by branches of the femoral nerve, obturator nerve, sciatic nerve, and lateral femoral cutaneous nerve. The femoral nerve, obturator nerve, and lateral femoral cutaneous nerve are all part of the lumbar plexus, implying that blocking the lumbar plexus is an elegant way to provide postoperative analgesia for THA [8]. However, lumbar plexus blocks may be associated with significant complications, including spinal and epidural injection, psoas hematoma or abscess, retroperitoneal hematoma, renal trauma, and systemic local anesthetic toxicity. The performance of this technique requires considerable expertise and it may be time-consuming to perform, thereby limiting its use. Recently, fascia iliaca block (FIB) has been recommended for the control of pain after THA [9]. However, despite being commonly used, there is conflicting evidence in recent reports regarding the efficacy of FIB in THA [10, 11]. In addition, due to their small sample sizes, these studies were not adequately powered to detect the effect of FIB on patients undergoing THA.

The primary aim of this systematic review and meta-analysis was to compare postoperative pain, analgesic consumption, and complications after FIB versus control for patients undergoing THA. Second, we compared the outcomes of FIB versus placebo. Finally, we sought to evaluate pain and analgesic consumption after preoperative and postoperative FIB.

## Methods

We followed the recommendations of the Cochrane Handbook for Systematic Reviews of Interventions [12] to carry out this study. We followed the PRISMA (Preferred Reporting Items for Systematic Reviews and Meta-Analyses) Guidelines to report our meta-analysis [13, 14]. The study protocol was registered with PROSPERO (registration number CRD42020212063).

### Search strategy

We performed a systematic literature search in MEDLINE, Embase, Scopus, Web of Science, Google Scholar, ClinicalTrials.gov, and CENTRAL through February 2021 to identify relevant studies with the assistance of a reference librarian with expertise in systematic review searches. The keywords “hip arthroplasty” or “hip replacement” and “fascia iliaca block,” “fascia iliaca blockade,” or “fascia iliaca*” were combined. Two reviewers independently screened the titles and abstracts of the identified studies for eligibility. References of the included studies were screened, and backward citation tracking was performed using Web of Science to identify articles not found in the original literature search.

### Selection criteria

After title and abstract screening, two reviewers independently reviewed the full-text articles. The inclusion criteria were primary THA; FIB versus control; reporting pain, analgesic consumption, or complication rate; and RCT. The exclusion criteria were revision THA, hip hemiarthroplasty, no availability of full text articles, observational studies, letters, meeting proceedings, and case reports. We had no inclusion restrictions based on the type of control cohort (placebo, lumbar plexus block, local anesthetic infiltration, spinal morphine, or patient-controlled intravenous analgesia [PCIA]). Disagreements about the eligibility of the full-text articles were resolved by consensus or by discussion with a third reviewer.

### Data extraction

Two reviewers extracted data independently using a predefined data extraction file. The following baseline characteristics were extracted from the included studies: first author, year of publication, study design, intervention, control, sample size, mean age, sex, time of administration, adjunct therapy in all patients, surgical operation, study type, and outcome data. Disagreements about the extracted data were resolved by consensus or by discussion with a third reviewer. Studies reporting on patient cohorts described in previously published articles were excluded or merged. The primary outcome measures were postoperative pain score (visual analog scale pain score [VAS] and numeric rating scale score [NRS]) at 24 h, and postoperative opioid consumption during the first 24 h. The secondary outcome measures were postoperative pain score in the PACU (0-2 h) and at 6 and 12 h, postoperative opioid consumption during the first 48 h, and complication rate. Complications included reports of anesthetic toxicity, neuropathy, hematoma formation, perforation, and opioid side effects.

### Quality assessment

Two reviewers used the Cochrane Risk of Bias tool [12] to assess the risk of bias in the RCTs. Each trial was reviewed and scored as high, low, or unclear risk of bias according to the following domains: random sequence generation, allocation concealment, blinding of participants and personnel, blinding of outcome assessment, incomplete outcome data, selective reporting, and other bias. Discrepancies between the reviewers were resolved by discussion until consensus was achieved.

### Statistical analysis

We decided a priori to perform meta-analyses when at least three studies with at least 100 patients per treatment arm were identified. We extracted dichotomous variables as absolute number and percentage, pooled them using the Mantel-Haenszel method, and presented them as risk ratio (RR) with 95% confidence interval (CI). We pooled continuous outcomes with the inverse variance weighting method and presented them as standard mean difference (SMD) with 95% CI. All analyses were conducted on intent-to-treat data with a random-effects model. We assessed the statistical heterogeneity among the studies by the I^2^ statistics for heterogeneity. We defined the significance level for treatment effects in primary and secondary outcomes as a P value less than 0.05. The quality of evidence across the statistically pooled outcomes in this meta-analysis was evaluated using the guidelines created by the Grades of Recommendation, Assessment, Development, and Evaluation (GRADE) [15]. We used Review Manager (RevMan, version 5.3.5) for all statistical analyses. We further assessed the publication bias with Begg’s and Egger’s statistical tests using Stata 13.1.

### Primary sensitivity analysis

We performed primary sensitivity analyses for postoperative pain at 24 h, analgesic consumption during the first 24 h, and complications with studies using placebo as a control cohort. We performed an additional sensitivity analysis for postoperative pain at 24 h, analgesic consumption during the first 24 h, and complications, with studies performing preoperative and postoperative FIB.

### Secondary sensitivity analyses

We performed secondary sensitivity analyses for high quality studies and different surgical approaches (traditional posterolateral approach or direct anterior approach) regarding postoperative pain at 24 h, analgesic consumption during the first 24 h, and complications. We performed additional sensitivity analyses for adjunct therapy (general anesthesia or spinal anesthesia), type of technique (ultrasound guided or fascial pop technique for FIB), and type of FIB (suprainguinal or classic FIB location).

### Trial sequential analysis

We conducted a trial sequential analysis (TSA) of the included studies using our primary outcome (pain at 24 h). TSA is a form of sequential hypothesis testing that analyzes the available data in chronological order. In meta-analyses, TSA can be used to assess the likely influence of future trials on the pooled findings and estimate the point at which further studies are not likely to change the pooled findings. To calculate the required information size (RIS) in this meta-analysis, the threshold for pain difference was set as more than 2, type 1 error was set to 5%, statistical power was set at 80%, and the estimated variance and heterogeneity were set from those present in the included trials [16]. We constructed the trial sequential analysis boundaries based on the O’Brien-Fleming alpha-spending function. We used trial sequential analysis software (version 0.9β, Copenhagen Trial Unit, Copenhagen, Denmark) to perform this analysis.

## Results

### Literature search and study characteristics

**Fig. 1:**
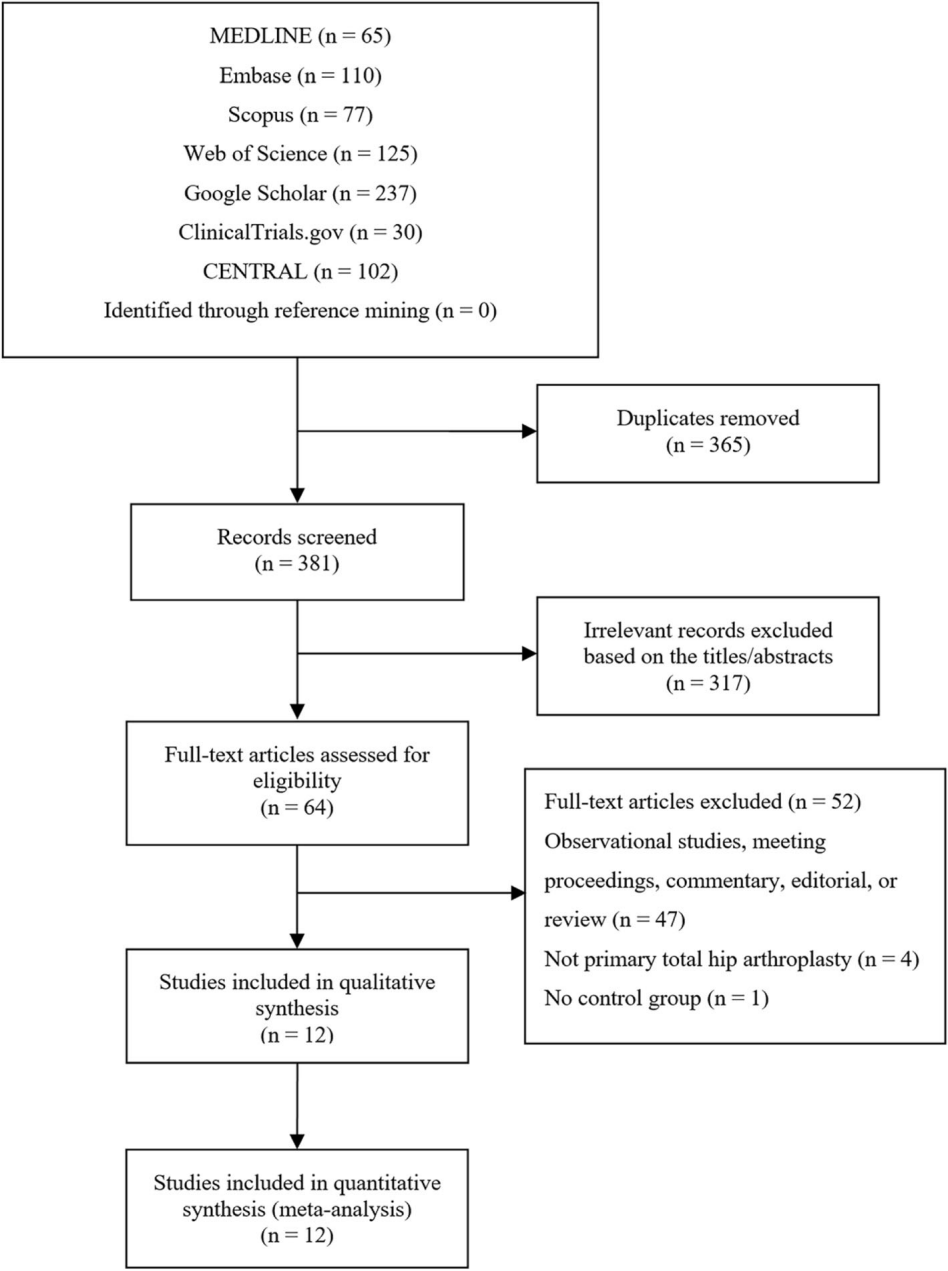
PRISMA flow diagram representing search and selection of studies comparing FIB versus control for THA

**Fig. 2:**
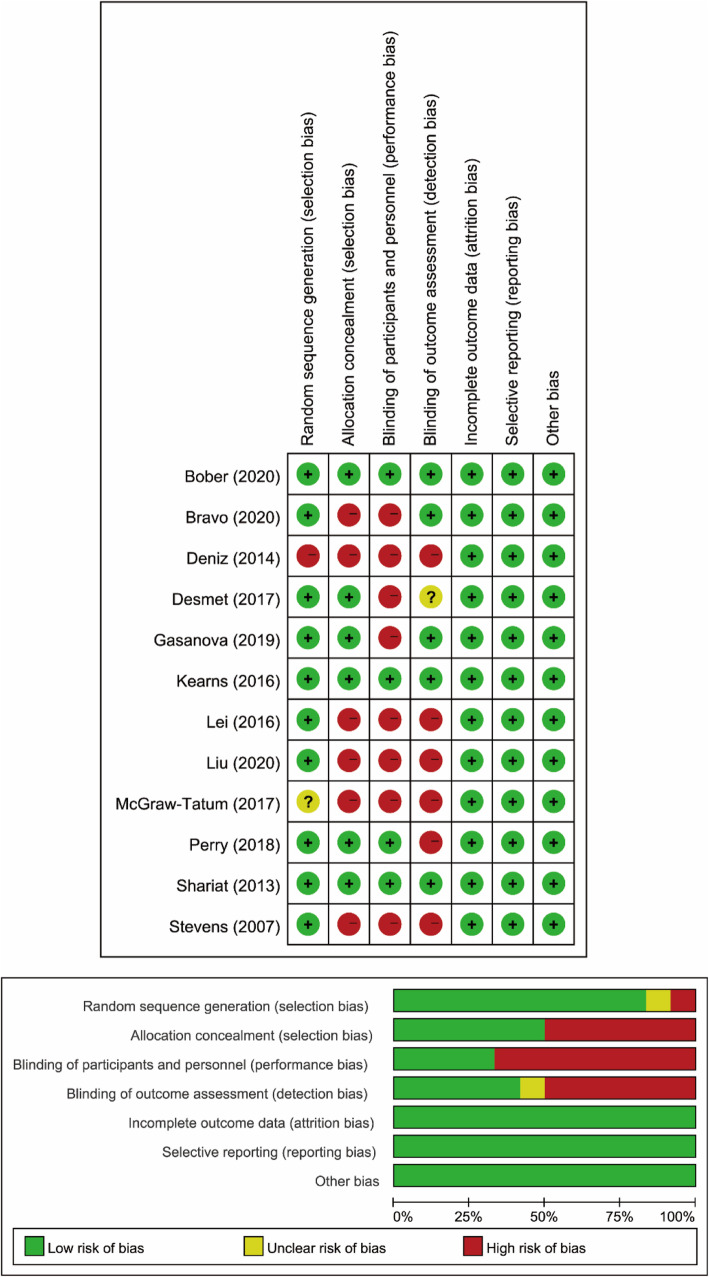
Risk of bias summary: review authors’ judgments about each risk of bias item for each included study. +, low risk of bias; −, high risk of bias; ?, unclear risk of bias

The search of the literature in different databases identified 746 articles. A total of 381 articles were evaluated after duplicates from each database were excluded. After screening the titles and abstracts, 64 articles were included, and full texts were assessed for eligibility. A total of 12 RCTs met our eligibility criteria: 6 RCTs compared outcomes of FIB versus placebo [10, 17–21], 2 RCTs compared outcomes of FIB versus periarticular infiltration [11, 22], 2 articles compared outcomes of FIB versus lumbar plexus block [9, 23], 1 article compared outcomes of FIB versus spinal morphine [24], and 1 article compared outcomes of FIB versus PCIA [25]. A total of 815 patients were included in this meta-analysis. Among the 12 RCTs, 2 RCTs [9, 21] used the intrathecal opioid, and 7 RCTs [9–11, 18, 22–24] used the multimodal analgesia for all patients. Descriptive study characteristics are shown in Table 1. A flowchart of the literature search is provided in Fig. 1. Among the 12 studies, three studies [10, 20, 24] were judged to be at low risk of bias, while nine [9, 11, 17–19, 21–23, 25] were found to have a high risk of bias (Fig. 2).

**Table 1:** Characteristics of the included studies

Author (year)	Intervention	Control	Sample size (Mean age, year)		Time of administration	Adjunct therapy in all patients	Study type
			Intervention	Control			
Bober (2020) [10]	FIB (40 mL of 0.25% bupivacaine)	Without FIB	60 (62.9)	62 (63.6)	Postoperative	Epidural lidocaine	RCT
Bravo (2020) [9]	FIB (40 mL of levobupivacaine 0.25% with epinephrine 5 μg/mL)	LPB (40 mL of levobupivacaine 0.25% with epinephrine 5 μg/mL)	30 (62.9)	30 (59.5)	Postoperative	Spinal anesthesia	RCT
Liu (2020) [19]	FIB (30 mL of 0.2% ropivacaine)	Without FIB	40 (70.1)	40 (70.0)	Postoperative	GA	RCT
Gasanova (2019) [22]	FIB (60 mL of ropivacaine 300 mg and epinephrine 150 μg)	Periarticular infiltration (60 mL of ropivacaine 300 mg and epinephrine 150 μg)	30 (56.2)	30 (59.0)	Postoperative	GA	RCT
Perry (2018) [23]	FIB (50 mL of 0.3% ropivicaine)	LPB (50 mL of 0.3% ropivicaine)	25 (58.9)	25 (58.1)	Preoperative	GA	RCT
McGraw-Tatum (2	FIB (40 mg of 0.2% ropivacaine)	Periarticular infiltration (20 mL of 1.3% liposomal bupivacaine)	39 (63.4)	40 (63.9)	Postoperative	GA	RCT
Desmet (2017) [18]	FIB (40 mL of 0.5% ropivacaine)	Without FIB	44 (60.4)	44 (66.5)	Preoperative	GA	RCT
Kearns (2016) [24]	FIB (40 mL of levobupivacaine, 2 mg.kg^−1^)	Spinal morphine (100 μg morphine)	54 (67)	54 (64)	Preoperative	Spinal anesthesia	RCT
Deniz (2014) [17]	FIB (30 mL of 0.25% bupivacaine)	Without FIB	24 (59.1)	22 (62.2)	Preoperative	GA	RCT
Shariat (2013) [20]	FIB (30 mL of 0.5% ropivacaine)	Without FIB	16 (61)	16 (57)	Postoperative	GA	RCT
Stevens (2007) [21]	FIB (30 mL 0.5% bupivacaine with 1:200,000 adrenaline, 150 μg clonidine)	Without FIB	22 (68.7)	22 (66.8)	-	Spinal anesthesia	RCT
Lei (2016) [25]	FIB (0.2% ropivicaine, 5 mL/h plus a bolus of 5 mL with a lock-time of 15 min)	PCIA (180 mL of tramadol, 0.3 mg/[kg·h], at a rate of 2 mL/h plus a bolus of 0.5 mL with a lock-time of 15 min)	23 (80.4)	23 (82.5)	Preoperative	GA	RCT

*FIB* fascia iliaca block, *LPB* lumbar plexus block, *GA* general anesthesia, *PCIA* patient-controlled intravenous analgesia, *RCT* randomized controlled trial

### Primary outcome measure

#### Postoperative pain at 24 h

**Fig. 3:**
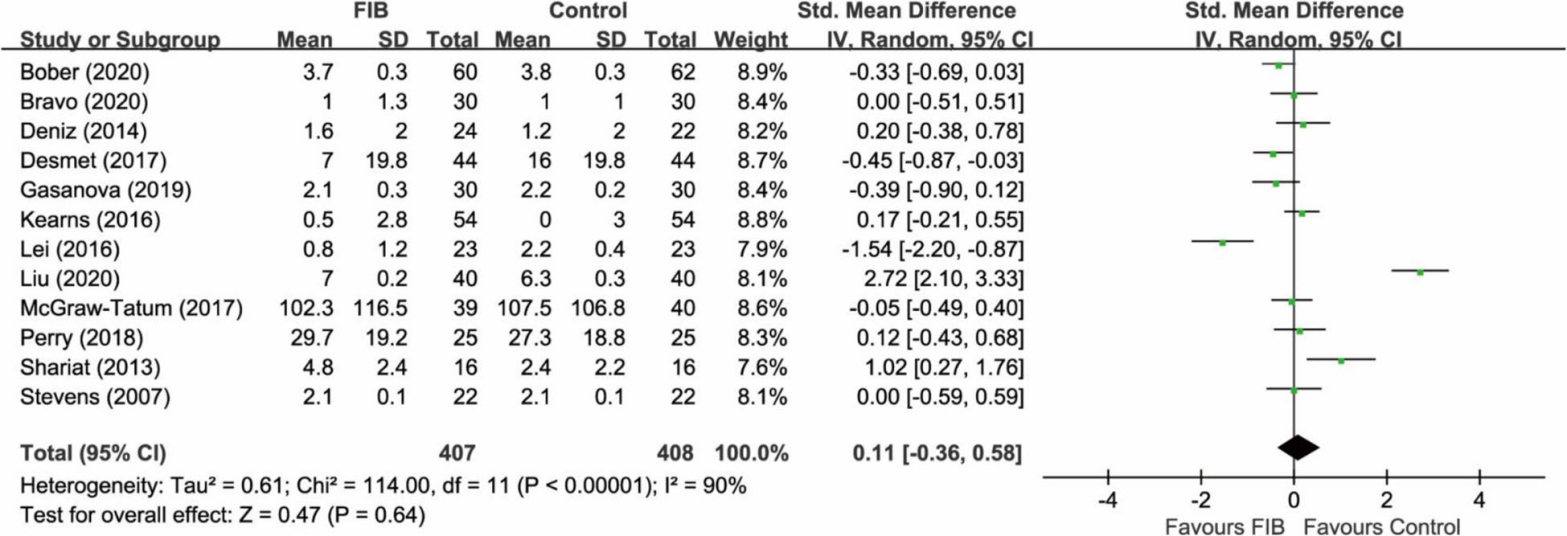
Forest plot of comparison: FIB versus control. Outcome: pain relief at 24 h. FIB, fascia iliaca block

A total of 12 RCTs (815 participants) [9–11, 17–25] provided relevant data on the postoperative pain at 24 h. The pooled analysis showed that FIB was associated with a similar pain relief at 24 h compared with the control group (SMD 0.11, 95% CI −0.36 to 0.58; P = 0.64) (Fig. 3). Heterogeneity was significant in the pooled result (I^2^ = 90%).

#### Postoperative opioid consumption during the first 24 h

**Fig. 4:**
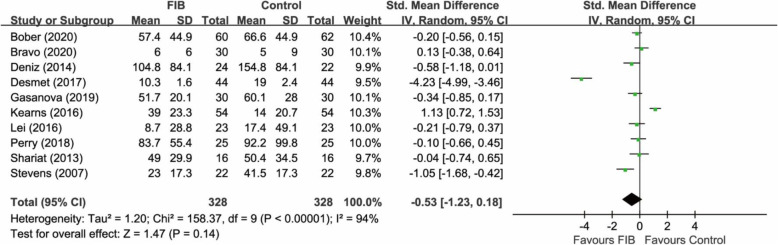
Forest plot of comparison: FIB versus control. Outcome: analgesic consumption during the first 24 h. FIB, fascia iliaca block

A total of 10 RCTs [9, 10, 17, 18, 20–25] (656 participants) provided relevant data on the postoperative opioid consumption during the first 24 h. The pooled analysis showed that FIB was associated with a similar postoperative opioid consumption during the first 24 h compared with the control group (SMD −0.53, 95% CI −1.23 to 0.18; P = 0.14) (Fig. 4). Heterogeneity was significant in the pooled result (I^2^ = 94%).

### Secondary outcome measure

#### Postoperative pain at 0-2 h

A total of 6 RCTs (398 participants) [10, 17, 18, 20, 22, 23] provided relevant data on the postoperative pain at 0-2 h. The pooled analysis showed that FIB was associated with a similar pain relief at 0-2 h compared with the control group (SMD −0.06, 95% CI −0.44 to 0.32; P = 0.76, I^2^ = 71%).

#### Postoperative pain at 6 h

A total of 6 RCTs (440 participants) [9, 10, 21, 22, 24, 25] provided relevant data on the postoperative pain at 6 h. The pooled analysis showed that FIB was associated with a similar pain relief at 6 h compared with the control group (SMD 0.22, 95% CI −0.21 to 0.65; P = 0.32). Heterogeneity was significant in the pooled result (I^2^ = 79%).

#### Postoperative pain at 12 h

A total of 7 RCTs (520 participants) [9, 10, 19, 21, 22, 24, 25] provided relevant data on the postoperative pain at 12 h. The pooled analysis showed that FIB was associated with a similar pain relief at 12 h compared with the control group (SMD 0.14, 95% CI −0.48 to 0.76; P = 0.65). Heterogeneity was significant in the pooled result (I^2^ = 91%).

#### Postoperative opioid consumption during the first 48 h

A total of 5 RCTs (438 participants) [9, 10, 18, 22, 24] provided relevant data on the postoperative opioid consumption during the first 48 h. The pooled analysis showed that FIB was associated with a similar postoperative opioid consumption during the first 48 h compared with the control group (SMD −0.62, 95% CI −1.85 to 0.62; P = 0.33). Heterogeneity was significant in the pooled result (I^2^ = 97%).

#### Complications

**Fig. 5:**
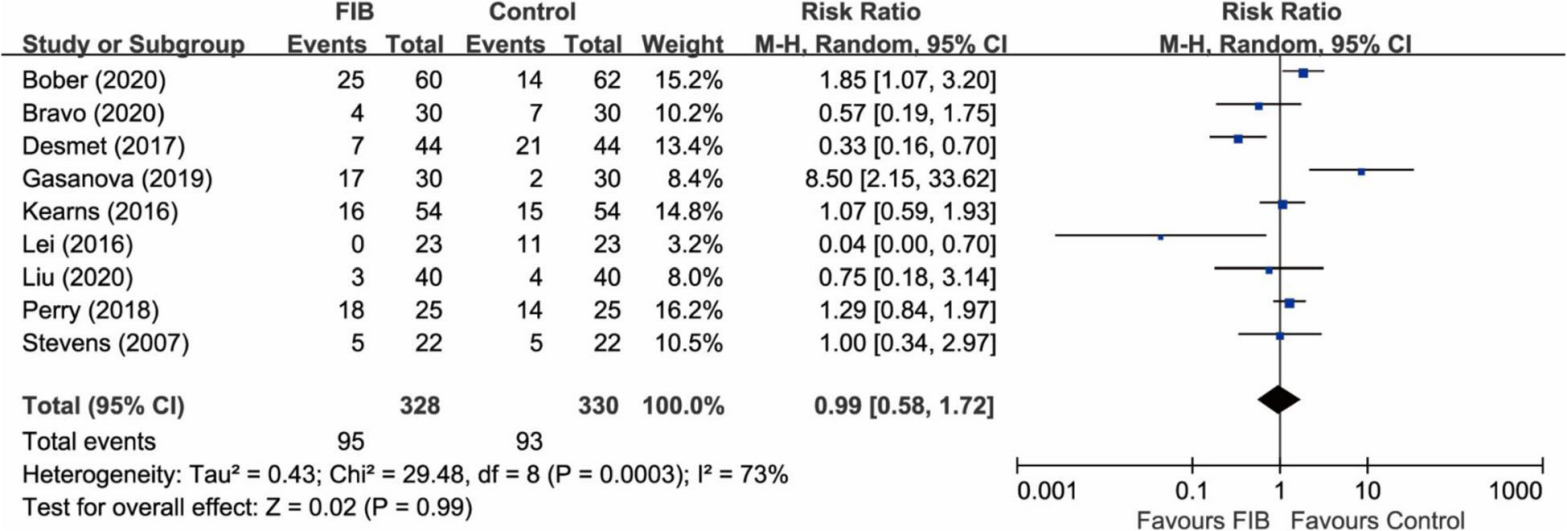
Forest plot of comparison: FIB versus control. Outcome: complication rate. FIB, fascia iliaca block

A total of 9 RCTs (658 participants) [9, 10, 18, 19, 21–25] provided relevant data on the complication rate. The pooled analysis showed that FIB was associated with a similar complication rate compared with the control group (RR 0.99, 95% CI 0.58 to 1.72; P = 0.99; I^2^ = 73%) (Fig. 5). Complications occurred in 30.0% of patients in the FIB group compared with 28.2% in the control group (risk difference 1.8%). The main complication after FIB was nausea and vomiting, which occurred in 13.4% of patients, followed by sensory changes (5.2%) and quadriceps weakness (4.0%). The main complication in the control group was also nausea and vomiting, which occurred in 16.7% of patients, followed by pruritus (3.0%) and dizziness (2.4%).

### Primary sensitivity analysis

#### FIB versus placebo

FIB versus placebo was reported in 6 RCTs [10, 17–21]. The overall pooled effect showed that there was no significant difference in pain relief at 24 h (SMD 0.51, 95% CI −0.38 to 1.39; P = 0.26; I^2^ = 94%), opioid consumption during the first 24 h (SMD −1.20, 95% CI −2.45 to 0.05; P = 0.06; I^2^ = 96%), or complication rate (RR 0.84, 95% CI 0.33 to 2.12; P = 0.71; I^2^ = 78%) between FIB and placebo.

#### Preoperative and postoperative FIB

Preoperative FIB was reported in 5 RCTs [17, 18, 23–25]. The overall pooled effect showed that there was no significant difference in pain relief at 24 h (SMD −0.27, 95% CI −0.82 to 0.27; P = 0.33; I^2^ = 83%), opioid consumption during the first 24 h (SMD −0.78, 95% CI −2.31 to 0.75; P = 0.32; I^2^ = 97%), or complication rate (RR 0.62, 95% CI 0.25 to 1.57; P = 0.31; I^2^ = 84%) between FIB and control.

Postoperative FIB was reported in 6 RCTs [9–11, 19, 20, 22]. The overall pooled effect showed that there was no significant difference in pain relief at 24 h (SMD 0.47, 95% CI −0.37 to 1.31; P = 0.27; I^2^ = 94%), opioid consumption during the first 24 h (SMD −0.14, 95% CI −0.38 to 0.10; P = 0.24; I^2^ = 0%), or complication rate (RR 1.59, 95% CI 0.59 to 4.28; P = 0.36; I^2^ = 71%) between FIB and control.

### Secondary sensitivity analyses

The results of the secondary sensitivity analyses are presented in Supplement Table 1. Secondary sensitivity analyses based on high quality studies, surgical approach (traditional posterolateral approach or direct anterior approach), adjunct therapy (general anesthesia or spinal anesthesia), type of technique (ultrasound guided or fascial pop technique for FIB), and type of FIB (suprainguinal or classic FIB location) were performed for postoperative pain at 24 h, opioid consumption during the first 24 h, and complications. The findings of postoperative pain at 24 h, analgesic consumption during the first 24 h, and complications were consistent in all secondary sensitivity analyses except for the suprainguinal FIB location group. In the suprainguinal FIB location group, we found that FIB was associated with a significantly higher pain relief than control at 24 h (SMD −0.30, 95% CI −0.57 to −0.03; P = 0.03; I^2^ = 0%).

### Trial sequential analysis

**Fig. 6:**
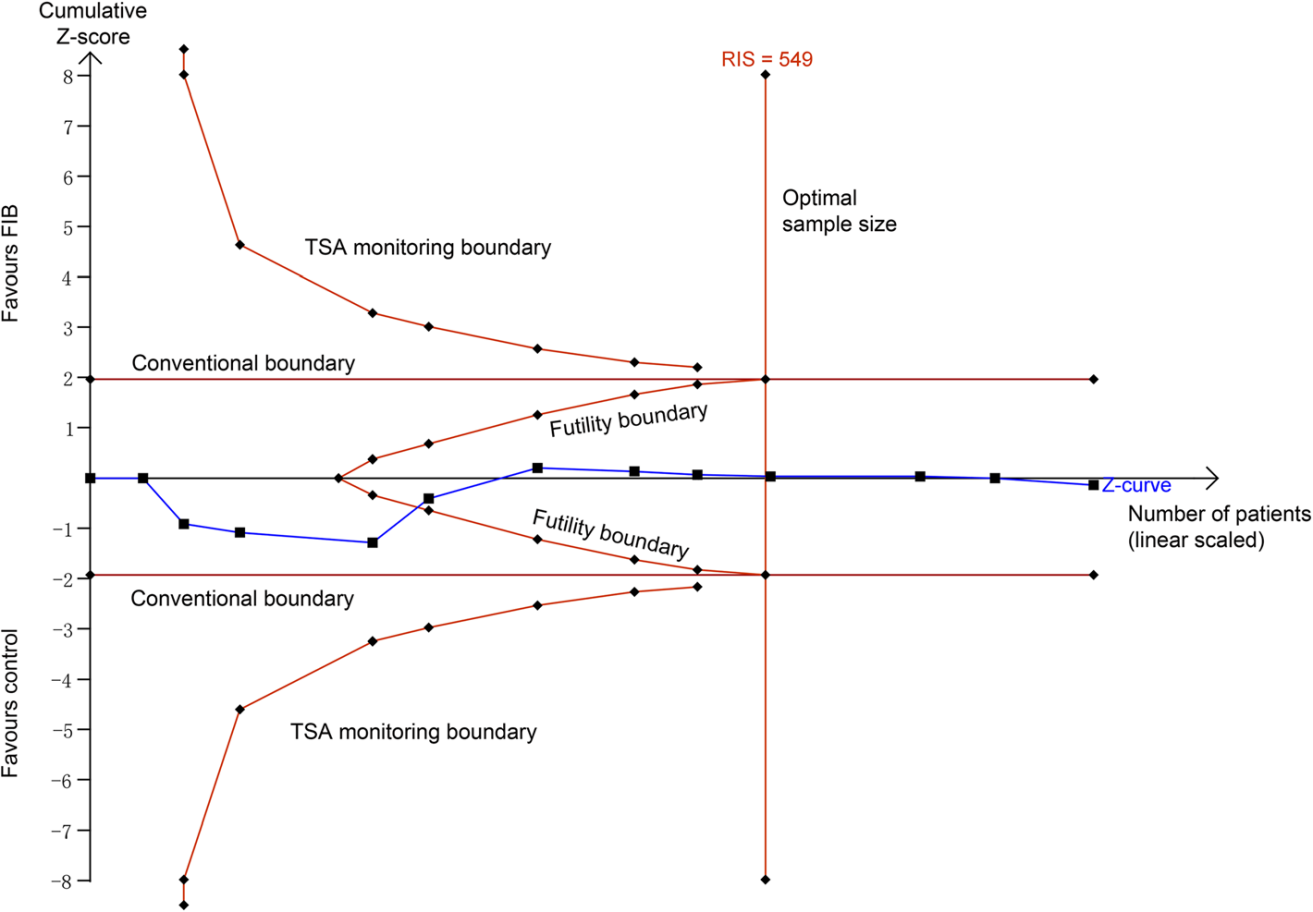
Trial-sequential analysis of 12 trials comparing FIB with control for postoperative pain at 24 h. The blue line represents the summary of what has been found after each trial, the Z score resulting from the cumulative evidence. The curved red lines are the TSA monitoring boundaries, for benefit (on the top of the graph), harm (on the bottom of the graph), and futility (the inner wedge). The horizontal red lines represent conventional model boundary of p < 0.05. The RIS is the required information size, which is an estimate of the number of participants required to answer the defined question. FIB, fascia iliaca block; RIS, required information size; TSA, trial-sequential analysis

For postoperative pain at 24 h, the cumulative Z score curve did not reach the 95% CI for statistical significance or the TSA monitoring boundaries. In addition, the Z score curve crossed the inner wedge of futility, while the total sample size exceeded the RIS (sample size = 815, RIS = 549) (Fig. 6). This indicates that additional studies are not likely to alter the conclusion that there is no significant difference in terms of pain at 24 h between the two cohorts.

### Publication bias

Egger and Begg’s tests were performed to investigate publication bias. The Egger’s test indicated no evidence of publication bias (P = 0.353). Similarly, in Begg’s test, there was no evidence of substantial publication bias (P = 0.193) (Supplement Figure 1).

## Discussion

This systematic review and meta-analysis identified 12 RCTs that compared the efficacy of FIB versus control for patients undergoing primary THA. The main findings of this review are summarized in Table 2. There was no difference in postoperative pain at 0-2 h, 6 h, 12 h, or 24 h; analgesic consumption during the first 24 h and 48 h; or complication rate between the FIB and control groups. Moreover, no difference in postoperative pain at 24 h, analgesic consumption during the first 24 h, or complication rate was observed between FIB and placebo. The GRADE rating for the main outcomes varied from low to moderate (Table 2).

**Table 2:** Summary of findings

Outcomes	Trials, n	Patients, n		Effect size (95% CI)	P value	I^2^ (%)	Quality of the evidence (GRADE)
		FIB	Control				
Postoperative pain at 0-2 h	6	199	199	−0.06 (−0.44, 0.32)	0.76	71	⨁⨁⊝⊝ low^ab^
Postoperative pain at 6 h	6	219	221	0.22 (−0.21, 0.65)	0.32	79	⨁⨁⊝⊝ low^ab^
Postoperative pain at 12 h	7	259	261	0.14 (−0.48, 0.76)	0.65	91	⨁⨁⊝⊝ low^ab^
Postoperative pain at 24 h	12	407	408	0.11 (−0.36, 0.58)	0.64	90	⨁⨁⨁⊝ moderate^a^
Postoperative opioid consumption during the first 24 h	10	328	328	−0.53 (−1.23, 0.18)	0.14	94	⨁⨁⨁⊝ moderate^a^
Postoperative opioid consumption during the first 48 h	5	218	220	−0.62 (−1.85, 0.62)	0.33	97	⨁⨁⊝⊝ low^ab^
Complications	9	328	330	0.99 (0.58, 1.72)	0.99	73	⨁⨁⨁⊝ moderate^a^

Reasons for downgrading the evidence level

^a^Heterogeneity was found

^b^Small sample bias may exist

*FIB* fascia iliaca block; *CI* confidence interval

THA is one of the most successful orthopedic operations performed for intractable hip pain due to primary and secondary osteoarthritis, osteonecrosis, and rheumatoid arthritis. It is estimated that over 300,000 total hip replacements are performed each year in the USA alone. In European countries, the number of hip replacement procedures performed in 2007 varied from fewer than 50 to more than 250 per 100,000 people [26]. Postoperative pain is one of the major concerns for patients undergoing THA. Controlling pain after hip replacement improves patient comfort and satisfaction and enables patients to participate in rehabilitation more fully, leading to an earlier return home and a reduced demand for resources. However, the use of postoperative opioid analgesia could increase the risk of opioid-related complications and delayed discharge. Several studies have shown that regional anesthesia could enhance recovery for THA [27]. The current meta-analysis aimed to investigate the FIB because it has several advantages over other lower extremity blocks, such as ease of performance and its track record of efficacy in the treatment of pain from hip fractures [28] and hip arthroscopy [29].

Our study contradicts the conclusion of previous systematic reviews. In two previous meta-analyses of 7 RCTs comparing FIB and control in patients undergoing THA, Cai et al. [30] and Gao et al. [31] found that FIB was associated with significantly better pain relief and lower morphine consumption after THA. However, there were several important methodological errors when they pooled the results. For example, although the measures of pain-related outcomes varied in the included studies (in the studies by Stevens et al. [21] and Shariat et al. [20], NRS was used; in the study by Deniz et al. [17], Lei et al. [25], and Desmet et al. [18], VAS was used), the authors took all of these outcome measures as VAS scores and pooled them together using weighted mean differences instead of standard mean differences. A similar error was also made when they pooled the morphine consumption data. Additionally, there were also several errors made when they selected the studies. For example, although these meta-analyses only included RCTs, the authors mistakenly classified an observational study [32] as an RCT, and included it in the analysis. Moreover, although the aim of these previous meta-analyses was to explore the effect of FIB for THA, the authors mistakenly included a study that only included patients with hemiarthroplasty [33]. Overall, both previous meta-analyses had obvious flaws that might threaten the authenticity of their findings. Compared with the previous meta-analyses with 7 studies and a total of 326 patients, the present systematic review and meta-analysis added 7 new RCTs which are the latest to date in this area. With a total of 815 patients, it provided the most comprehensive update on the efficacy of FIB for THA. Only RCTs were included; therefore, by excluding observational studies, we removed the inherent selection bias associated with that study design.

Our study suggested that FIB did not provide significant analgesia after THA, which may be because of incomplete coverage of the surgical field. It is well known that the femoral and obturator nerves innervate the anteromedial and anterolateral portions of the hip, respectively. However, posteriorly, the sciatic nerve, the nerve to the quadratus femoris, and the superior gluteal nerve are responsible for the superior, inferior, and lateral portions of the joint [8]. These nerves originate in the sacral plexus and are not covered by FIB, which may be the reason why FIB was not found to be superior to placebo or different anesthetic techniques.

Some limitations of our study need to be mentioned. First, there is substantial heterogeneity in the adjunct therapy (general anesthesia, spinal anesthesia, and epidural lidocaine), type of technique (ultrasound guided or fascial pop technique for FIB), type of FIB (suprainguinal or classic FIB location), and surgical approach for THA (direct anterior approach or traditional posterolateral approach). Although we performed sensitivity analyses for these potential influencing factors, the studies included in each analysis were limited (many were less than 5 studies). These have weakened our inference of the effect of FIB compared with control for THA. Additionally, the included studies were rather heterogeneous in terms of their control cohorts. Although we performed sensitivity analyses for studies using placebo as a control cohort, and found no difference between FIB and placebo, for other control cohorts such as periarticular infiltration and spinal morphine, we did not perform sensitivity analyses for them due to the limited number of studies (≤ 2) included in the analysis. Moreover, several studies included in the analysis suffered from important methodological limitations. The potential risk of bias that those studies pose has weakened our inference of the treatment effects.

In conclusion, FIB was not found to be superior to placebo or various anesthetic techniques for patients undergoing primary THA, as measured by postoperative pain, analgesic consumption, and complications.

## Supplementary Information

**Additional file 1: eTable 1.** Secondary sensitivity analyses for postoperative pain at 24 hours, opioid consumption during the first 24 hours, and complications. **eFigure 1.** Test for publication bias. Results showed that evidence of publication bias was not found (p=0.193).

## Data Availability

The data are available from the corresponding author upon reasonable request.

## Abbreviations

FIB: Fascia iliaca block
THA: Total hip arthroplasty
RCTs: Randomized controlled trials
PCIA: Patient-controlled intravenous analgesia
VAS: Visual analog scale pain score
NRS: Numeric rating scale score
RR: Risk ratio
CI: Confidence interval
SMD: Standard mean difference
TSA: Trial sequential analysis
RIS: Required information size
