# Supplementary material for: The effect of fascia iliaca block on postoperative pain and analgesic consumption for patients undergoing primary total hip arthroplasty: a meta-analysis of randomized controlled trials
Source: J Orthop Surg Res. 2021 Jul 9;16:444. doi: 10.1186/s13018-021-02585-1 (PMC8268399; doi:10.1186/s13018-021-02585-1)
Supplement: Supplementary file 1 — Additional file 1: eTable 1. Secondary sensitivity analyses for postoperative pain at 24 hours, opioid consumption during the first 24 hours, and complications. eFigure 1. Test for publication bias. Results showed that evidence of publication bias was not found (p=0.193). [file 13018_2021_2585_MOESM1_ESM.docx]

**eTable 1.** Secondary sensitivity analyses for postoperative pain at 24 hours, opioid consumption during the first 24 hours, and complications.

| Groups^*^ | Postoperative pain at 24 hours | | | Opioid consumption during the first 24 hours | | | Complications | | |
| --- | --- | --- | --- | --- | --- | --- | --- | --- | --- |
|  | SMD (95% CI) | P | I^2^, % | SMD (95% CI) | P | I^2^, % | RR (95% CI) | P | I^2^, % |
| High quality studies | 0.21 (-0.42, 0.84) | 0.51 | 82 | 0.30 (-0.63, 1.23) | 0.52 | 92 | NA | NA | NA |
| Surgical approach | | | | | | | | | |
| Traditional posterolateral approach | NA | NA | NA | NA | NA | NA | NA | NA | NA |
| Direct anterior approach | 0.79 (-1.02, 2.60) | 0.39 | 97 | NA | NA | NA | 0.70 (0.24, 2.04) | 0.51 | 82 |
| Adjunct therapy | | | | | | | | | |
| General anesthesia | 0.23 (-0.68, 1.15) | 0.62 | 94 | -0.90 (-1.97, 0.17) | 0.10 | 95 | 0.85 (0.27, 2.63) | 0.78 | 84 |
| Spinal anesthesia | 0.09 (-0.18, 0.36) | 0.53 | 0 | 0.09 (-1.11, 1.29) | 0.89 | 94 | 0.91 (0.45, 1.84) | 0.79 | 0 |
| Type of technique | | | | | | | | | |
| Ultrasound guided FIB | 0.12 (-0.38, 0.63) | 0.64 | 91 | -0.47 (-1.22, 0.29) | 0.22 | 95 | 1.12 (0.47, 2.66) | 0.80 | 78 |
| Fascial pop technique for FIB | NA | NA | NA | NA | NA | NA | NA | NA | NA |
| Type of FIB | | | | | | | | | |
| Suprainguinal FIB | -0.30 (-0.57, -0.03) | 0.03 | 0 | -1.46 (-3.75, 0.82) | 0.21 | 98 | 1.10 (0.17, 6.86) | 0.92 | 89 |
| Classic FIB location | 0.25 (-0.37, 0.87) | 0.43 | 92 | -0.13 (-0.68, 0.41) | 0.63 | 87 | 1.15 (0.50, 2.64) | 0.73 | 56 |

FIB: fascia iliaca block; SMD: standard mean difference; RR: risk ratio; CI: confidence interval; NA: Not applicable.

^*^We did not perform sensitivity analysis for the results with limited number of studies (≤ 2) included in the analysis.


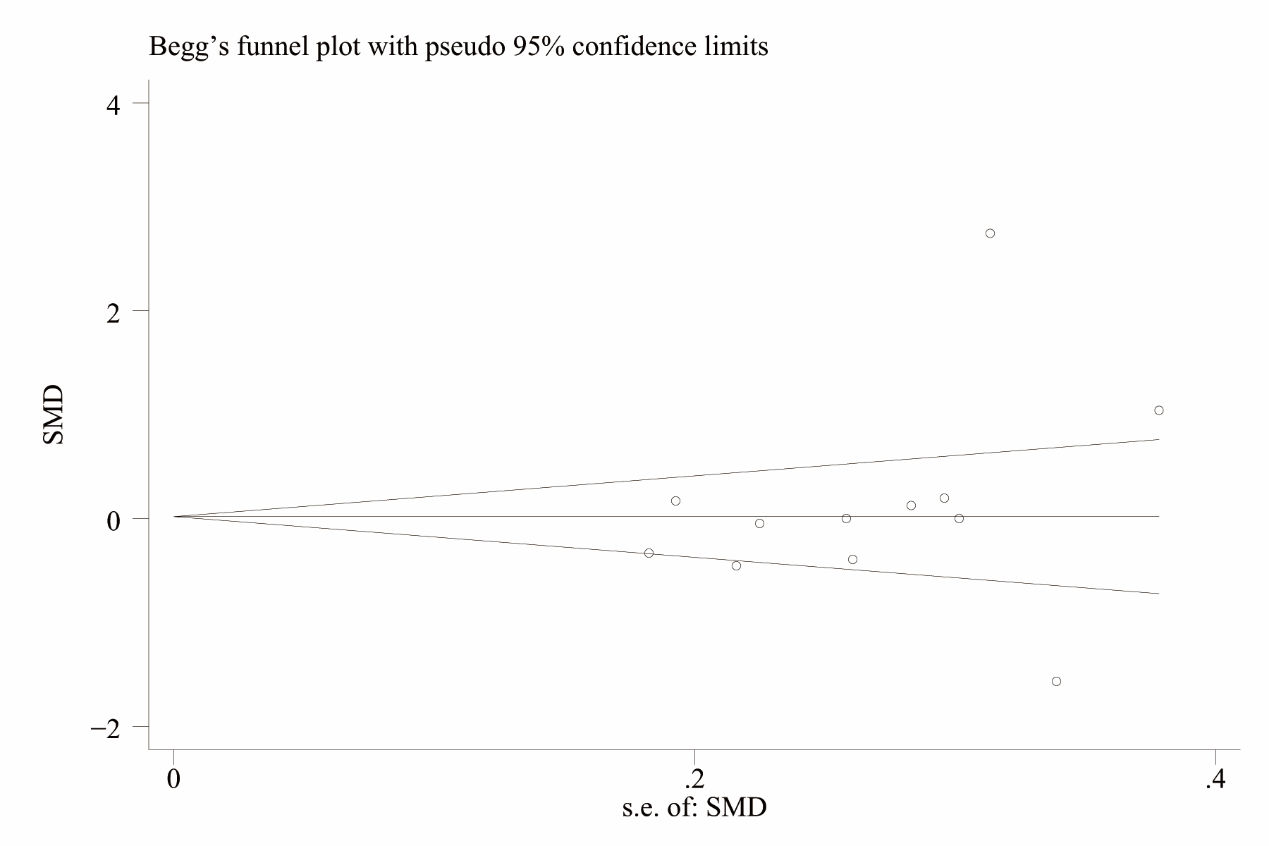


**eFigure 1.** Test for publication bias. Results showed that evidence of publication bias was not found (p=0.193).
